# The trend in mean height of Guatemalan women born between 1945 and 1995: a century behind

**DOI:** 10.1186/s41043-022-00324-8

**Published:** 2022-09-15

**Authors:** Astrid Arriaza, K. Michael Hambidge, Nancy F. Krebs, Ana Garcés, Andrew Amos Channon

**Affiliations:** 1 Institute of Nutrition of Central America and Panama, Calzada Roosevelt, 6-25, Zona 11, Guatemala City, 01011 Guatemala; 2 University of Colorado School of Medicine, 12700 East 19th Avenue, Box C225, Aurora, CO 80045 USA; 3 Centre for Global Health and Policy, University of Southampton, Southampton, UK

**Keywords:** Height trend, Inequality, Socio-economic development

## Abstract

**Background:**

Adult height is a cumulative indicator of living standards with mean height increasing with a greater socio-economic level. Guatemalan adult women have the lowest mean height worldwide. The country’s population is ethnically divided between indigenous and non-indigenous groups. This study aims to identify trends in the mean height for indigenous and non-indigenous adult women born between 1945 and 1995 in Guatemala and the association with individual, household and environmental factors.

**Methods:**

We used pooled data of adult women from five Demographic and Health Surveys. Mixed-effects multilevel linear regression models estimate the mean height associated with the explanatory variables. Mean height was modelled as a function of birth year cohort, wealth, education, geo-administrative regions and elevation.

**Results:**

The mean height increased 0.021 cm per year on average. The annual increase for indigenous women was 0.027 cm, while 0.017 cm for non-indigenous women. Height is associated with household wealth and women’s education level. We found an interaction effect between ethnicity and household wealth, with indigenous women at the lowest quintile 0.867 cm shorter than the corresponding non-indigenous group. Height is associated with the geo-administrative region, those women in western regions being shorter than those in the metropolis. Mean height is reduced 0.980 cm for each 1000 m increase in elevation.

**Conclusions:**

Guatemalan women have grown only 1 cm over half century, a slow improvement between 1945 and 1995, a period characterised by political instability and civil war. There are persistent inequalities in women’s height associated with socio-economic status, education and attributes of the geographical context. These aspects need to be considered when implementing strategies to encourage growth. Further research is required to understand the evolution of adult height and the standard of living in post-war Guatemala.

## Background

Mean adult height can be used as a cumulative indicator of social conditions and the standard of living of a population [1, 2]. The secular trend of mean adult height of a population is frequently used to explore the evolution or increase in social conditions, including social and economic development over a period of time [1, 3, 4]. Attained adult height is influenced by diverse factors related to human growth at different developmental stages [2]. These determinant factors are classified at three analytical levels: individual, household and environmental level, providing a conceptual framework to explore differences in adult height [2, 3].

The individual level contains biological factors of human growth, including biochemical factors genetics, nutrition and health [5–8]. An optimal nutrition enables post-natal growth [5, 9, 10], while ill-health episodes experienced during childhood can have a negative effect on nutrient absorption [3]. Individual-level factors associated with growth are likely to be interrelated with household-level factors [11]. Previous research identified social and economic factors observed at household level associated with differences in adult height [2, 12, 13]. Increased mean adult height is observed among women of higher socio-economic status [14, 15]. The environmental factors consider the social and physical conditions in which people live, including cultural, macroeconomic and political factors [7]. Differences in adult height can be observed in relation to historical events such as famine, specific socio-economic periods and the psychosocial context [4, 16, 17].

The secular trend of adult height can be related to the social development experienced by the society in a given time period [1]. The secular trend explores the annual rate of change, providing evidence about the magnitude of the height growth over time. Previous research has identified that the mean adult height increased in the twentieth century across the world, some countries experiencing an increase of 20 cm during this period [13]. This trend has been related to the improvement of the standard of living in industrialised and post-industrialised societies [3, 18, 19], with expected differentials between regions [13] and within countries [14, 15]. Aside from providing information linked with social progress, exploring the secular trend of adult height can provide evidence about social inequalities.

Guatemala has the lowest mean height for adult women in the world [13]. Model-based estimates of adult height in the twentieth century identified the mean height of Guatemalan women born in 1896 which was 140.3 cm, with a 9 cm increase over one hundred years [13]. Scholars focusing on the anthropometric history of Latin America in the second half of the twentieth century found that Guatemala was one of the countries with the lowest height increase in the region [20]. Similar to findings in other countries, adult height in Guatemala has been associated with socio-economic status [15]. Scholars have found that the height gap between women at the lowest and the highest socio-economic group in Guatemala is one of the widest out of 54 low- and middle-income countries (LMICs) [15].

Guatemala has been historically characterised by high levels of socio-economic inequalities, being one of the countries with the lowest public expenditure for social development programmes in Latin America [21]. The proportion of the population living in relative poverty has remained similar over recent decades, with 59.3% of the population classified as being below the poverty line, while 23.4% are under extreme poverty in 2014 [22]. Guatemala is an ethnically diverse country, ranking second with the highest proportion of indigenous population in the region [23]. Greater socio-economic disadvantages have been observed among indigenous populations, with 79.2% of its population classified as below the poverty line [22]. Other ethnic differences have been observed in relationship with social capital and labour opportunities [21, 24].

Poverty and poor standards of living in Guatemala have been associated with child growth deficit [25, 26]. Results from the latest Demographic and Health Surveys in 2014–2015, estimates that 46.5% of children were stunted [27], positioning Guatemala among the top ten countries for child stunting in the world [28]. Child stunting in Guatemala has been associated with different factors including ethnic background, children born to an indigenous mother more likely to have a low height-for-age compared to non-indigenous children [25; 29–32]. Considering the high prevalence of child stunting, the ethnic divide and being the shortest female adult population in the world, it becomes relevant to explore differences in adult height between indigenous and non-indigenous women in Guatemala.

We hypothesise that there will be a difference in height between indigenous and non-indigenous population groups mediated by socio-economic factors measured at individual and household level, as well as environmental-level factors. Previous research conducted in Guatemala has shown economic and social inequalities related to human growth at early age, and we explore how these factors are related to height among Guatemalan women. The aim of this research is to identify differences in the mean adult height for indigenous and non-indigenous women over a 50 years period, women born between 1945 and 1995, a period characterised by political turmoil ending in 1996 with the peace agreements [33] and the implementation of Neo-liberal economic policies [34].

## Methods

### Data

The height trend analysis used individual-level data from five Guatemalan Demographic and Health Surveys (DHS) [27, 35–38]. The DHS are a cross-sectional survey that collects data from a nationally representative sample. The sample selection follows a two-stage cluster sampling procedure; the first stage is the selection of primary sampling units (PSUs) with probabilities proportional to population size, by urban and rural population and socio-economic stratification; the second stage represents the systematic selection of households within each PSU. This research used pooled data from the 1995, 1998–1999, 2002, 2008–2009 and 2014–2015 DHS. The DHS collect individual data from women between 15 and 49 years old including the woman’s height at the time of the interview.

The analytical sample was restricted to individual records having a valid height measurement. The DHS programme has used two different strategies for recording women’s height across the years. DHS, prior to 2003, recorded woman’s height only for those women having a child under the age of 5 years at the time of the survey using the same questionnaire. The following surveys have used independent questionnaires allowing height measurements for every women in the household [39]. The pooled sample size for women with height measurement is 57,169 women between 15 and 49 years old.

The sample size was further limited to women aged 20 years and older; this age value was used in order to include only those women that had stopped growing. Women with extreme height values were excluded by applying a cut-off value of five standard deviations from the mean. The sample was reduced after removing women who had missing values for any of the explanatory variables. The final sample size was *n* = 45,043 women, aged between 20 and 49 years old at the time of the interview with a height measurement (Table 1).

**Table 1:** Sample size of adult women extracted from Guatemalan Demographic and Health Surveys (DHS), 1995–2015

Sample population	1995	1998–1999	2002	2008–2009	2014–2015	Total
Woman with height measurement (15–49 years)	5811	2736	8105	16,326	24,191	57,169
Women with height measurement (20–49 years)	5501	2704	7023	14,087	19,909	49,224
Women with height measure without missing values (20–49 years)	5102	2604	6970	10,460	19,907	45,043

Data source: DHS 1995, DHS 1998–1999, DHS 2002, DHS 2008–2009 and DHS 2014–2015 [27, 35–38]

### Outcome variable

The women’s individual height at the time of the interview is the outcome variable used in this analysis. Height is measured in centimetres, with an accuracy of 0.1 cm, by trained personnel following standardised DHS procedures [40].

### Explanatory variables

The pooled data allow characterisation of the height trend over the 50-year period. The secular trend models height as a function of the year of birth, with additional explanatory variables used to model the mean height. Ethnicity, categorised as indigenous or non-indigenous, was used to control for individual-level factors. An index to measure the relative household wealth and the woman’s attained education level was used as a proxy indicator of household-level determinants. The relative wealth index is a composite value created from assets, services and amenities variables and derived by principal component analysis. This household index is used as a proxy variable of socio-economic status and living standards [41]. The attained education level was used as a social class indicator. Environmental-level factors included place of residence (urban/rural), geo-administrative region and elevation of the sampling cluster for every 1000 m above the sea level.

### Statistical analysis

Adult height was modelled as function of birth year cohort, including other explanatory variables. This study includes exploratory bivariate and multivariate analysis. The bivariate analysis was conducted prior to modelling the trend in height to compare mean height difference. These differences and the explanatory variable groups were tested using t tests and one-way analysis of variance. Multicollinearity among the explanatory variables was checked using the variance inflation factor.

Multivariate analysis was conducted to test the association between height and the selected explanatory variables. This analysis used generalised linear models to account for the nested structure of the sampling procedure. We applied mixed-effects linear regression models, with women (level 1) nested within PSUs (level 2). Fixed effects describe regression coefficients at level one, while the random effects at level two identify variance between clusters. Sample weights were applied to obtain unbiased estimates.

The analysis used three models: one for the overall sample and two independent models for the ethnic groups. The first model estimates the mean height for adult women using a random coefficient model, allowing the coefficient to vary between ethnic groups. We tested for interactions between household wealth quintiles, location and ethnicity to assess the independence of these variables. Explanatory variables were included by a forward stepwise selection, and the best model fit was selected using the Akaike information criterion (AIC) and log-likelihood function. The two-level linear mixed-effects regression model describing the overall height trend is written as follows:

$$y _{ij} = \beta_{0} + \beta_{1} x_{1ij} + \ldots + \beta_{n} x_{nij} + \left( {u_{1j} x_{1ij} + u_{0j} + e_{ij} } \right)$$

where ${y }_{ij}$ is the height in cm of the *ith* woman within the *jth* PSUs, ${\beta }_{o}$ is the overall mean height for a woman in the reference categories, ${\beta }_{1}\dots {\beta }_{n}$ are the fixed coefficients, and ${x}_{nij}$ is the vector of covariates corresponding to these coefficients for the *ith* woman within the *jth* PSUs. Random effects are given by$\left({{u}_{1}x}_{1ij}+{u}_{oj}+{e}_{ij}\right)$, where ${{u}_{1}x}_{1ij}$ is the random effect introduced for ethnicity, ${u}_{0j}$ is the random effect of the PSUs, and ${e}_{ij}$ is the residual term for every woman. The random effects${u}_{0j}$, ${u}_{1j}$ and ${e}_{ij}$ follow a normal distribution with mean zero, and we assume$Var \left({u}_{0j}\right)= {\sigma }_{u0}^{2},Var \left({u}_{1j}\right)= {\sigma }_{u1}^{2} , Cov \left({u}_{0j}, {u}_{1j}\right)= {\sigma }_{u01} and Var \left({e}_{ij}\right)= {\sigma }_{e}^{2}$.

Two additional models identify the secular trend independently by ethnic group. The independent models use a multilevel model strategy to fit a two-level linear regression. The variables were included by a forward stepwise selection. Model fit was assessed by AIC and log-likelihood function, while variance partition coefficient (VPC) was used to estimate the unexplained variability at the second level. The association between women’s height and the covariates is expressed in cm and the corresponding standard error (SE).

Other frameworks have conceptualised human development, including growth, by a social–economic–political–emotional framework, in which the material and moral conditions of the society might have an effect on human development [42]. Elements of this have been included in our model, which are possible through the DHS surveys. This analysis considered that there might be periods during the five decades for which the annual growth rate might differ. Changes in the speed of the height trend are hypothesised in relationship with disruption of regular activities during the 36 years civil conflict. To test this hypothesis, the linear model included splines in the regression, to identify a nonlinear relationship for given periods in time. This analysis assumed that the annual growth rate was different before the civil war, between 1945 and 1959, and during the conflict, between 1960 and 1995 [33]. The findings of this analysis identified no significant effect of the splines, and therefore, the simple linear regression approach was chosen instead.

## Results

This research identified the mean height for indigenous and non-indigenous adult woman born in Guatemala between 1945 and1995, alongside factors associated with the mean height. The mean height for women born in Guatemala between 1945 and1995 was 148.8 cm, (95% CI, ± 0.371). The mean height for an indigenous woman during this period was 146.0 cm (95% CI, ± 0.453) and 150.8 cm, (95% CI, ± 0.422) for non-indigenous women. This research found statistically significant differences for mean height according to the ethnic group, attained education level, household wealth index, place of residence and geo-administrative region (Table 2).

**Table 2:** Mean height of Guatemalan women born between 1945 and 1995

Variable	Category	*n*	%	Mean ht (cm)	± 95% CI	*p* value^1^
Population sample		45,043	100.0	148.83	0.371	
Ethnicity	Non-indigenous	26,825	59.6	150.75	0.422	< 0.0001
	Indigenous	18,218	40.4	145.98	0.453	
Highest education level	None	9367	20.8	145.57	0.000	< 0.0001
	Primary	25,627	56.9	148.63	0.604	
	Secondary	8201	18.2	151.90	0.799	
	Higher	1848	4.1	154.04	1.778	
Wealth quintile	Lower	9319	20.7	145.97	0.606	< 0.0001
	Low	10,333	22.9	147.08	0.611	
	Middle	10,028	22.3	149.00	0.714	
	High	8167	18.1	150.43	0.611	
	Higher	7196	16.0	153.09	0.876	
Location	Urban	18,349	40.7	150.28	0.592	< 0.0001
	Rural	26,694	59.3	147.85	0.437	
Region	Metropolitan	4929	10.9	151.53	1.078	< 0.0001
	Central	5954	13.2	148.88	0.995	
	North	3987	8.9	147.73	1.094	
	Northeast	6446	14.3	150.75	0.999	
	Southeast	4751	10.5	150.67	1.079	
	Northwest	4993	11.1	145.80	0.873	
	Southwest	11,660	25.9	147.42	0.610	
	Petén	2323	5.2	149.66	1.488	

^1^*T* test and one-way analysis of variance

Data source: DHS 1995, DHS 1998–1999, DHS 2002, DHS 2008–2009 and DHS 2014–2015 [27, 35–38]

On average, the mean height for women born in Guatemala between 1945 and 1995 increased 0.021 cm annually (Table 3). For the overall model, we have found a negative within cluster-level correlation between mean height and the differences in height between indigenous and non-indigenous women (*r* = − 0.64), indicating that clusters with a shorter overall mean tend to have a smaller height gap between indigenous and non-indigenous women (Table 3). Further analysis found that 17.7% and 15.6% of the total variability in height are due to variation between clusters for indigenous and non-indigenous women, respectively.

**Table 3:** Variables associated with the mean height of women born in Guatemala between 1945 and 1995

Variable	Categories	Coefficient	SE	*p* value^1^
	Intercept	147.0	0.246	< 0.001
Annual increase	Year birth cohort	0.021	0.003	< 0.001
Ethnicity	Non-indigenous	Reference		
	Indigenous	− 0.867	− 0.867	< 0.001
Education level	None	Reference		
	Primary	0.995	0.073	< 0.001
	Secondary	2.547	0.098	< 0.001
	Higher	3.762	0.141	< 0.001
Wealth quintile	1st	Reference		
	2nd	1.302	0.142	< 0.001
	3rd	2.229	0.143	< 0.001
	4th	3.167	0.149	< 0.001
	5th	4.913	0.163	< 0.001
Location	Urban	Reference		
	Rural	0.157	0.189	
Geo− administrative region	Metropolitan	Reference		
	Central	− 0.750	0.204	< 0.001
	North	− 0.349	0.242	
	Northeast	− 0.107	0.221	
	Southeast	0.772	0.223	< 0.001
	Northwest	− 1.168	0.224	< 0.001
	Southwest	− 1.272	0.184	< 0.001
	Petén	− 0.100	0.317	
Elevation	per 1,000 m above sea level	− 0.979	0.073	< 0.001
Ethnicity * Wealth quintile	2th * Indigenous	− 0.759	0.179	< 0.001
	3th *Indigenous	− 1.316	0.188	< 0.001
	4th * indigenous	− 1.428	0.201	< 0.001
	5th *indigenous	− 2.250	0.231	< 0.001
Random effects	Intercept variance	3.092		
	Ethnicity variance	5.093		
	Residual variance	15.47		
	Correlation coefficient	− 0.64		

^1^Mixed-effects linear regression model

Data source: DHS 1995, DHS 1998–1999, DHS 2002, DHS 2008–2009 and DHS 2014–2015 [27, 35–38]

**Fig. 1:**
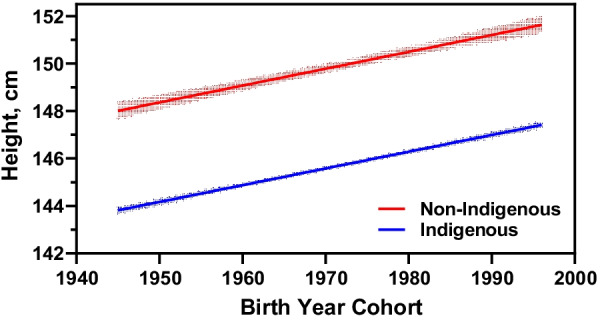
Mean height trend for indigenous and non-indigenous women born in Guatemala between 1945 and 1995. The annual height increase is different for indigenous and non-indigenous women with mean height for indigenous women remaining lower than for non-indigenous women across the period of analysis. On average, the height gap was 4.77 cm (95% CI, ± 0.382)

The models by ethnic group show differences in the annual growth rate for indigenous and non-indigenous women (Table 4). This research found that indigenous women had an increase of 0.027 cm per year during the 50-year period of analysis. This value is higher compared to the corresponding annual increase for non-indigenous woman, increasing 0.017 cm per year. Despite the higher annual growth rate, indigenous women’s mean height remained lower than the mean height for non-indigenous women throughout the period of study. The mean height trend for indigenous and non-indigenous women is illustrated in Fig. 1.

**Table 4:** Variables associated with the mean height for indigenous and non-indigenous women born in Guatemala between 1945 and 1995

Variable	Categories	Indigenous women			Non-indigenous women		
		Coefficient	SE	*p* value^1^	Coefficient	SE	*p* value^1^
	Intercept	146.42	0.339	< 0.0001	146.81	0.296	< 0.0001
Annual increase	Year birth cohort	0.027	0.004	< 0.0001	0.017	0.004	< 0.0001
Education level	None	Reference			Reference		
	Primary	0.736	0.095	< 0.0001	1.341	0.114	< 0.0001
	Secondary	2.096	0.144	< 0.0001	2.984	0.138	< 0.0001
	Higher	2.887	0.249	< 0.0001	4.332	0.182	< 0.0001
Wealth quintile	Lower	Reference			Reference		
	Lowest	0.557	0.109	< 0.0001	1.274	0.143	< 0.0001
	Middle	0.931	0.128	< 0.0001	2.151	0.145	< 0.0001
	High	1.753	0.151	< 0.0001	3.03	0.152	< 0.0001
	Highest	2.890	0.201	< 0.0001	4.721	0.168	< 0.0001
Geo− Admin. Region	Metropolitan	Reference			Reference		
	Central	− 1.245	0.309	< 0.0001	− 0.343	0.25	
	North	− 0.874	0.324	< 0.001	0.514	0.354	
	Northeast	0.246	0.382		− 0.068	0.251	
	Southeast	1.097	0.398	< 0.001	0.844	0.245	< 0.0001
	Northwest	− 1.692	0.305	< 0.0001	− 0.253	0.317	
	Southwest	− 1.379	0.274	< 0.0001	− 1.207	0.226	< 0.0001
	Petén	− 0.972	0.496	< 0.01	0.353	0.384	
Elevation	per 1,000 m above sea level	− 0.971	0.495	< 0.0001	− 0.998	0.103	< 0.0001
Random effects	Intercept variance	3.188			2.932		
	Residual variance	14.827			15.883		
	Variance Partitioning Coefficient	17.77%			15.61%		

^1^Mixed-effects linear regression model

Data source: DHS 1995, DHS 1998–1999, DHS 2002, DHS 2008–2009 and DHS 2014–2015 [27, 35–38]

**Fig. 2:**
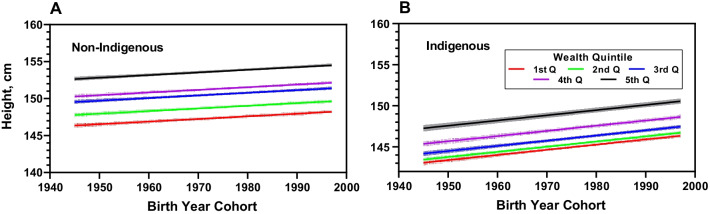
Mean height trend by wealth quintile for non-indigenous **A** and indigenous **B** women born in Guatemala between 1945 and 1995. Women’s mean height is associated with household socio-economic status (SES) with a significant interaction effect between ethnicity and household wealth. Data extracted from [27, 35–38]

Women’s mean height is associated with the household socio-economic status. On average, women in the highest quintile were 4.91 cm (Table 3) taller than the lowest group (Fig. 2). Moreover, we found an interaction between ethnicity and household wealth (Table 3). Indigenous women from the lowest wealth quantile, on average, were 0.867 cm shorter than non-indigenous women in the same group (Table 3). Compared to non-indigenous women from the lowest wealth level, the height gap of an indigenous woman is increasingly negative as wealth level increases. This finding highlights that women’s height is not only biologically or ethnically determined, but also depends on socio-economic status. The gap in height between wealth quintiles for indigenous women is smaller than the gap for non-indigenous women. The mean height for indigenous women in the highest quintile was 2.89 cm taller than an indigenous woman from the first quintile, while the mean height for a non-indigenous woman was 4.72 cm taller (Table 4).

**Fig. 3:**
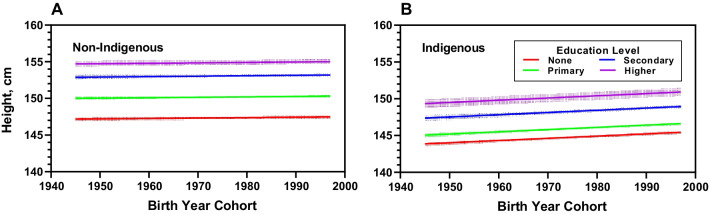
Mean height by educational level for non-indigenous **A** and indigenous **B** women born in Guatemala between 1945 and 1995. The mean height of Guatemalan women is associated with the attained education level; non-indigenous women at the highest education level had the greatest mean height. Data extracted from [27, 35–38]

Women that attained the highest education level were 3.76 cm taller (Table 3), than those with no formal education. The gap across educational levels for indigenous women is smaller than that across educational levels for non-indigenous women. The mean height for indigenous women who reached the highest education level was 2.89 cm taller than those without schooling, while the mean height for non-indigenous women was 4.33 cm (Table 4). Figure 3 illustrates the mean by educational level for indigenous and non-indigenous women separately.

Height is associated with environmental-level factors. On average, women living in the Southwest and Northwest regions at the time of the interview were 1.27 cm and 1.17 cm, respectively, shorter than women from the metropolitan region (Table 3). Mean height differs across geo-administrative regions for indigenous and non-indigenous women. Indigenous women living in the metropolitan region have a higher mean height than indigenous women living in the rest of the regions, except for the Southeast region (Table 4). Non-indigenous women had smaller differences in height between regions, although those living in the Southwest region were 1.21 cm shorter than their counterparts living in the metropolitan region. Height decreases with higher elevation; we found a reduction of 0.98 cm per 1,000-m increase in elevation above the sea level, on average (Table 3). This reduction is seen for indigenous and non-indigenous populations (Table 4). No association was observed between urban/rural location and height in Table 3 after adjusting for household wealth.

## Discussion

This research explored the annual increase in height for Guatemalan indigenous and non-indigenous women over fifty years. Guatemalan women have increased, on average, 0.21 cm per decade, representing an increase of 1 cm over 50 years. This annual growth can be considered slow when compared to other Latin American countries over the same period. Latin American countries experiencing economic modernisation during this period, such as Brazil, Mexico, and Colombia, have experienced a mean height gain of 0.9 cm per decade [20]. This growth increase is also lower than a number of LMICs [15] and smaller than the growth rate in the nineteenth century in Europe [18]. Considering that adult height can be used as a cumulative indicator of the standard of living, the reduced annual increase in the mean height of Guatemala women highlights the slow development of the standard of living that this population experienced between 1945 and 1995.

The reduced annual height in the overall population of women is accompanied by an overall short adult height across the country. Using the results from the model coefficients, the predicted mean height for women born in Guatemala in the year 2000 was 150.8 cm. This predicted height is comparable in magnitude with the standard height of an 11-year-old girl proposed by the World Health Organization [43] and similar to the mean height of a Colombian woman born at the beginning of the twentieth century [44]. This research highlights the presence of ethnic inequalities in height over the past half century. Despite identifying a faster growth rate for indigenous women, they remain significantly shorter than non-indigenous women. Mean height differences between ethnic groups are associated with household wealth, with the biggest differential between ethnicities seen among the wealthiest. These findings imply that women’s height is not only ethnically or individually determined, rather is related to the socio-economic status.

Adult height inequalities associated with the ethnic background during this period can potentially be explained by differentiated social development and cultural experiences. There is some evidence about greater social and economic disadvantage among indigenous populations, having higher probability of primary school dropout compared non-indigenous children [24]. Previous scholars have identified that indigenous women have experienced discrimination at public health care services, a perception likely to influence health care-seeking experiences [45, 46]. These experiences can potentially have a negative impact in the use of maternal and child health care services among indigenous women [47]. Social disadvantages among indigenous population are also observed by the increased proportion of stunted children [25, 29–32].

The difference in the rate of annual height increase by ethnicity suggests differentiated evolution in the standard of living. The differentiated development can be observed by the evolution of some social determinant factors over the last five decades, including cultural, educational and labour changes. Cultural changes among indigenous population can be exemplified by the decline in the proportion of women wearing indigenous traditional clothes or by the decline of women speaking a Mayan language; these are indicators of ethnic identity [48, 49]. Educational changes are being observed by the increased proportion of indigenous children enrolled at primary school [23] and the declining ethnic gap in mathematics achievement [24, 50]. Labour changes have also been observed with a reduced proportion of indigenous population employed in agriculture [23] and by the shift from traditional agricultural production towards export production [51, 52]. Researchers have identified that the agricultural production change has improved the standard of living and increased the households food expenditure [53]. However, there is no consensus about the effect of the agrarian production change and the effect on child nutrition [54].

This research identified an increased mean height associated with a higher education level and at higher socio-economic status, similar to research conducted in other counties. Education might be an indicator of increased capacity for the assimilation of ideas; previous research conducted in Guatemala has identified positive hygiene beliefs [55] and increased likelihood for seeking formal medical care at increased levels of education [47]. The positive effect of wealth on adult height can potentially be related to access to sanitation, health care and diet. Previous research exploring access of health care services for children experiencing diarrhoea is strongly associated with family income [56]. Scholars have identified that wealthier families are more likely to have a diet with an increased protein intake, while poorest families are more likely to have a traditional diet based on maize and beans [57]. Maize and the traditional diet have a relevant role in Mayan culture [58]. However, given the historical period, it is likely to be associated with a less diverse diet among poorest groups [59, 60].

This research found that women living in the Western regions are shorter than women from the metropolis. This difference can potentially be explained by differences in social characteristics and costs to access nutritional foods in these regions. The Southwest and Northwest are characterised by a high proportion of rurality and indigenous population [23]. In contrast, the metropolitan area has a higher proportion of individuals classified at middle and upper socio-economic status as well as reduced proportion of indigenous population [61]. Previous research conducted in 1969 measuring geographic accessibility to food identified that the Western regions have increased costs to access highly nutritional foods, while the Southeast region offered better accessibility [62].

This research found that an increase in elevation is associated with a decrease in mean adult height. This finding was also identified in research exploring child stunting in Guatemala [25] and in other countries [63]. A greater elevation might be linked with greater difficulties for food production given the ecology of the area, steep land, susceptibility to erosion and reduced moisture absorption [64]. These environmental factors are relevant conditions that require adaptations of the food production in agrarian societies [65]. Living at higher elevation in Guatemala has been considered an indicator of reduced access to land, public services, and from the historical perspective, mountainous areas have contributed to ethnic division during colonial times [66]. Further research is required to explain the relationship between human growth and the environmental conditions at high altitude and possible confounding factors.

This research has a number of limitations; the study is a cross-sectional study using data collected at one single point in time measured at adulthood, limiting the understanding of previous circumstances of the individuals. The socio-economic information collected represents an observation at the point of time when the growth period has finished and might not be representative of the social conditions during childhood. Hence, cautious interpretation of these results is required. Despite this limitation, the women’s attained education level and the household socio-economic status at adulthood can be considered a social class indicator, since these two factors are strongly determined by the family social status [67] and supported by the low socio-economic mobility of the country at that time [61]. The analysis is limited by the data available; there might be other factors explaining the differences, including long exposure to violence [68]. However, the DHS do not provide data about violence at the individual level or political violence at the local level.

## Conclusions

Guatemala women have grown only 1 cm over a half century. The annual growth rate during this period is different for indigenous and non-indigenous women, with indigenous women having an increased rate. Inequalities in the mean height of adult woman are dependent on ethnicity, socio-economic status, education and geographical context. Previous research has indicated that human growth and development have an interconnected nature between biology and socio-economic status, and other factors [1, 2, 42]. Hence, improving aspects such as sanitation, living standards, nutrition and general health during development will aid in growth in Guatemala.

This research has identified a slow improvement of the standard of living for Guatemalan women born between 1945 and 1995. This period in history was characterised by social and political instability beginning in 1944 with the end of one of the most repressive dictatorships in Latin America and followed by a civil war that ended with the peace accords in 1996. The novel sociopolitical stability brought by the democratic era might allow a faster increase in adult height; researchers using nationally representative data have identified that child stunting between 1995 and 2014 has declined 9.4% [32]. Further research is required to understand the evolution of living standards and social development in post-war Guatemala as well as understanding the population health status in short height populations.

## Data Availability

The Guatemalan Demographic and Health Surveys (DHS) datasets used to generate results presented in the current study are available at: https://www.dhsprogram.com/Publications/Publication-Search.cfm?ctry_id=15&country=Guatemala (27, 35–38).

## Abbreviations

AIC: Akaike information criterion
CI: Confidence intervals
DHS: Demographic and Health Surveys
LMICs: Low- and middle-income countries
PSU: Primary sampling unit
SE: Standard error
VPC: Variance partitioning coefficient
